# Commentary: Distributed Cognition and Distributed Morality: Agency, Artifacts and Systems

**DOI:** 10.3389/fpsyg.2018.00490

**Published:** 2018-04-09

**Authors:** Witold M. Wachowski

**Affiliations:** ^1^Institute of Philosophy, University of Warsaw, Warsaw, Poland; ^2^Institute of Philosophy and Sociology, Polish Academy of Sciences, Warsaw, Poland

**Keywords:** distributed cognition, extended cognition, cognitive system, cognitive integration, cognitive interaction

Studies on human–artifact interaction stimulate reflection on the bounds of cognition, agency, and even morality (e.g., Floridi and Sanders, 2004). Heersmink (2017) compares distributed cognition (DCog) and distributed morality theory and claims that some artifacts, depending on their use, have cognitive and moral status but lack cognitive and moral agency. According to him, an extended cognitive system (ECS) has agency when artifact(s) included in the system are fully transparent and densely integrated into the cognitive processes of the user, whereas a distributed cognitive system (DCS) without central control lacks agency. My doubts do not concern Heersmink's main claim. Irrespective of the final assessment of the moral status of distributed systems, I argue that the assumption that the assessment of the degree to which humans and artifacts are cognitively integrated is not always feasible and distorts our understanding of DCog.

## Extension and distribution of the cognitive

Heersmink (2017) sums up the well-known concepts of “wide” cognition: cognitive states and processes may go beyond individual minds to involve people and artifacts; human agents and artifacts form integrated systems performing information-processing tasks. Cognitive activity sometimes extends beyond the brain to non-neuronal parts of the body and elements of the environment. Two famous examples are: a navigation team on board of a surface vessel at sea (Hutchins 1995), and a man with Alzheimer's disease who supports his biological memory by means of a notebook (Clark and Chalmers, 1998). As Heersmink writes: “Clark's extended cognition theory focuses on single agents interacting with artifacts, whereas Hutchins DCog theory typically (though not exclusively) focuses on larger systems with more than one agent interacting with artifacts. In such wider cognitive systems, there are thus one or more individuals interacting and coupling with cognitive artifacts” (Heersmink, 2017).

Heersmink recognizes the significant difference between the two concepts, relying on Hutchins's comments (Hutchins, 2014): extended cognition is just a special case of DCog opening up a much broader view of the different types of cognition. Among them, “[s]ome systems have a clear center while other systems have multiple centers or no center at all” (Hutchins, 2014, p. 37).

So far, Heersmink's summary is not controversial. What is problematic is his view that it “is better to conceive of system membership in terms of the degree of cognitive integration of humans and artifacts” (Heersmink, 2017). This integration depends on different dimensions including the kind and intensity of information flow between human and non-human components, accessibility of the scaffold, durability of the coupling, amount of user trust, degree of transparency-in-use, ease of interpretation of the information and the amount of personalization or cognitive transformation (Heersmink, 2015, 2017). Hence, cognitive artifacts can be integrated more or less deeply.

## Cognitive system as a mechanism

Let's go back to the way Hutchins defines DCSs. For him, distribution means interaction (Hutchins, 2006, p. 376–377). When we take the DCog perspective, we do not “make any claim about the nature of the world. Rather, it is to choose a way of looking at the world, one that selects scales of investigation such that wholes are seen as emergent from interactions among their parts” (Hutchins, 2014, p. 36). DCog is not a kind of cognition, but a perspective on all of cognition. “[T]he notions of centralized and distributed are always relative to some scale of investigation. (…) The boundaries of the unit of analysis for DCog are not fixed in advance; they depend on the scale of the system under investigation, which can vary (…)” (Hutchins, 2014, p. 36).

DCS—to which ECSs belong—doesn't constitute any more or less integrated agent “casing.” Hutchins shows that DCSs may have different scales (the brain is one example), and the large systems he studied offer something like Gulliver's perspective in the land of giants: an opportunity for direct observation of cognitive processes in the macroscale occurring in an environment (Hutchins, 1995, p. 128–129).

Therefore, the assumption that it is always possible to grade the cognitive integration between an agent and artifact may fail in the case of some complex DCSs. Artifacts are not “attached” to the “genuinely” cognitive part of the system but are its equally important components. It is only the system as such that can have agency potential or can be the center for something else.

DCS should be viewed as a mechanism (e.g., Bechtel and Abrahamsen, 2005; Ylikoski, 2015). A mechanism is a structure that performs a function by means of its organized component parts and operations. It is responsible for one or more phenomena. Mechanisms can occur in nested hierarchies and can work cyclically, but they can also be responsible for one-off events only. Mechanisms can also be computational (Miłkowski, 2013; Piccinini, 2015). Thus, in the DCS it is the interaction of active components and time coordination that is important for its mechanism(s), whilst the components themselves can be physically separated and coordinated only temporarily. At the same time, mechanisms may be more or less durable, and more or less tightly organized. In other words, Heersmink's claims can be better stated in the mechanistic framework without distorting the original idea of DCog.

## Concluding remarks

For Heersmink, an ECS has agency when an artifact is fully transparent and densely integrated into the cognitive processes of its user; for this reason (according to Heersmink's criterion for being an agent and having agency), a distributed system without central control lacks agency because it is not a system whose intentions are being realized. My doubts do not concern Heersmink's main claim, but a minor one, although important for understanding DCog. In complex cases, it is meaningless to ask about the degree of cognitive integration of humans and artifacts in DCSs. Demanding such an integration brings to mind the anthropomorphic fallacy. The ECS in which a person can fully control the operation of her/his artificial extensions is only a special simple case of DCog. Wider cognitive systems are not added to “genuine” cognitive systems, but they themselves are “genuine” systems: their components interact in a coordinated manner, as in the case of any mechanism.

## Author contributions

WW reviewed the literature and developed the theoretical stance and wrote the manuscript and prepared to publication.

### Conflict of interest statement

The author declares that the research was conducted in the absence of any commercial or financial relationships that could be construed as a potential conflict of interest.

## References

[B1] Bechtel W. Abrahamsen A. (2005). Explanation: a mechanist alternative. Stud. Hist. Philos. Biol. Biomed. Sci. 36, 421–441. 10.1016/j.shpsc.2005.03.010 19260199

[B2] Clark A. Chalmers D. (1998). The extended mind. Analysis 58, 7–19.

[B3] Floridi L. Sanders J. (2004). On the morality of artificial agents. Minds Mach. 14, 349–379. 10.1023/B:MIND.0000035461.63578.9d

[B4] Heersmink R. (2015). Dimensions of integration in embedded and extended cognitive systems. Phenom. Cogn. Sci. 14, 577–598. 10.1007/s11097-014-9355-1

[B5] Heersmink R. (2017). Distributed cognition and distributed morality: agency, artifacts and systems. Sci. Eng. Ethics 23, 431–448. 10.1007/s11948-016-9802-1 27380187

[B6] Hutchins E. (1995). Cognition in the Wild. Cambridge, MA: MIT Press.

[B7] Hutchins E. (2006). The distributed cognition. Perspective on human interaction, in Roots of Human Sociality: Culture, Cognition and Interaction, eds Enfield N. J. Levinson S. C. (Oxford: Berg Publishers), 375–398.

[B8] Hutchins E. (2014). The cultural ecosystem of human cognition. Philos. Psychol. 27, 34–49. 10.1080/09515089.2013.830548

[B9] Miłkowski M. (2013). Explaining the Computational Mind. Cambridge, MA: MIT Press.

[B10] Piccinini G. (2015). Physical Computation: A Mechanistic Account. Oxford: Oxford University Press.

[B11] Ylikoski P. (2015). Social mechanism, in International Encyclopedia of the Social & Behavioral Sciences, 2nd Edn. Vol. 22, ed Wright J. D. (Amsterdam: Elsevier), 415–420. 10.1016/B978-0-08-097086-8.03194-9

